# VHHs as tools for therapeutic protein delivery to the central nervous system

**DOI:** 10.1186/s12987-022-00374-4

**Published:** 2022-10-03

**Authors:** Yessica Wouters, Tom Jaspers, Laura Rué, Lutgarde Serneels, Bart De Strooper, Maarten Dewilde

**Affiliations:** 1 Present Address: VIB Center for Brain and Disease Research, Campus Gasthuisberg O&N4, Herestraat 49, box 602, 3000 Louvain, Belgium; 2 Laboratory for the Research of Neurodegenerative Diseases, Department of Neurosciences, Leuven Brain Institute (LBI), KU Leuven, 3000 Louvain, Belgium; 3 Present Address: Department of Biotechnology and Biomedicine, Technical University of Denmark, 2800 Kongens Lyngby, Denmark; 4 UK Dementia Research Institute, University College London, London, UK; 5 Present Address: Laboratory for Therapeutic and Diagnostic Antibodies, Department of Pharmaceutical and Pharmacological Sciences, KU Leuven, 3000 Louvain, Belgium

**Keywords:** Nanobody, VHH, Transferrin receptor, Neurotensin, Blood–brain barrier, Receptor-mediated transcytosis

## Abstract

**Background:**

The blood brain barrier (BBB) limits the therapeutic perspective for central nervous system (CNS) disorders. Previously we found an anti-mouse transferrin receptor (TfR) VHH (Nb62) that was able to deliver a biologically active neuropeptide into the CNS in mice. Here, we aimed to test its potential to shuttle a therapeutic relevant cargo. Since this VHH could not recognize the human TfR and hence its translational potential is limited, we also aimed to find and validate an anti-human transferrin VHH to deliver a therapeutic cargo into the CNS.

**Methods:**

Alpaca immunizations with human TfR, and subsequent phage selection and screening for human TfR binding VHHs was performed to find a human TfR specific VHH (Nb188). Its ability to cross the BBB was determined by fusing it to neurotensin, a neuropeptide that reduces body temperature when present in the CNS but is not able to cross the BBB on its own. Next, the anti–β-secretase 1 (BACE1) 1A11 Fab and Nb62 or Nb188 were fused to an Fc domain to generate heterodimeric antibodies (1A11AM-Nb62 and 1A11AM-Nb188). These were then administered intravenously in wild-type mice and in mice in which the murine apical domain of the TfR was replaced by the human apical domain (hAPI KI). Pharmacokinetic and pharmacodynamic (PK/PD) studies were performed to assess the concentration of the heterodimeric antibodies in the brain over time and the ability to inhibit brain-specific BACE1 by analysing the brain levels of Aβ_1–40_.

**Results:**

Selections and screening of a phage library resulted in the discovery of an anti-human TfR VHH (Nb188). Fusion of Nb188 to neurotensin induced hypothermia after intravenous injections in hAPI KI mice. In addition, systemic administration 1A11AM-Nb62 and 1A11AM-Nb188 fusions were able to reduce Aβ_1-40_ levels in the brain whereas 1A11AM fused to an irrelevant VHH did not. A PK/PD experiment showed that this effect could last for 3 days.

**Conclusion:**

We have discovered an anti-human TfR specific VHH that is able to reach the CNS when administered systemically. In addition, both the currently discovered anti-human TfR VHH and the previously identified mouse-specific anti-TfR VHH, are both able to shuttle a therapeutically relevant cargo into the CNS. We suggest the mouse-specific VHH as a valuable research tool in mice and the human-specific VHH as a moiety to enhance the delivery efficiency of therapeutics into the CNS in human patients.

**Supplementary Information:**

The online version contains supplementary material available at 10.1186/s12987-022-00374-4.

## Background

Drug development for central nervous system (CNS) related diseases has been more challenging compared to non-CNS indications. This is indicated by higher attrition rates during clinical trials, which often occur later in development, leading to the cost of CNS drug development to be amongst the highest of any therapeutic indication [1]. One of the reasons why CNS drug development is so challenging, is the presence of the blood–brain barrier (BBB), which restricts therapeutics from entering the CNS. This restricted access leads to the need of high peripheral doses to reach therapeutically relevant concentrations in the brain. As a result peripheral side effects might occur. Moreover lots of antibody have to be administered which makes the cost of the treatment enormous. These potential issues are especially of concern in major CNS-related diseases, such as Alzheimer’s or Parkinson’s disease, for which currently no cure exists. If a successful antibody would be generated for such frequent diseases, demand might rapidly exceed current available production capacities [2]. By making drug transport to the CNS more efficient, lower peripheral doses would be needed to reach relevant concentrations in the CNS and to obtain the desired therapeutic effects.

An example of a suitable method for long-term drug delivery to the CNS is drug delivery by receptor-mediated transcytosis (RMT), a transport mechanism which is also used by endogenous macromolecules to reach the brain. Here, therapeutics are targeted to receptors expressed at the BBB, leading to transport from the periphery to the CNS. Currently, monoclonal antibodies (mAbs) generated against two receptors, transferrin receptor (TfR) and insulin receptor (InsR), are known to be able to transport therapeutic proteins to the human brain [3, 4]. In animal models other antibody formats, such as single-chain variable fragments (scFvs) [5–7], Fab fragments [8], engineered Fc fragments [9, 10] and shark variable domain antibodies (VNAR) [11, 12], can deliver biologics to the CNS utilizing the TfR. Also affinity binders targeting CD98hc and anti-IGF1R were shown to reach the brain after peripheral injection [13, 14]. Recently, our group published the discovery of the first brain-penetrating anti-TfR VHH (Nb62) using the hypothermic effect of neurotensin (NT) as a CNS target-engaging readout [15]. A VHH is the variable domain isolated from heavy chain only antibodies originating from camelids, and is also known under the name ‘Nanobody™’ [16]. Just like VNARs, VHHs are known to be highly stable and can be used as building blocks to generate multispecific antibodies or antibody-enzyme fusions [16]. The latter is particularly useful in the context of BBB-shuttling moieties, as the shuttling moieties need indeed to be linked to therapeutic payloads. For VNARs, research is still limited to preclinical research and it is not known how they will behave in human, especially with respect to their potential immunogenicity. They originate from cartilaginous fish like sharks, and their sequential and structural homology towards human immunoglobulins is considerably lower compared to immunoglobulins originating from mammals (e.g. VHHs originating from camelids) [17]. Although humanization strategies have been proposed, it needs to be seen if this will be sufficient to avoid immunogenicity in patients [17, 18]. In contrast, the low immunogenicity and clinical potential of VHHs in patients have been demonstrated extensively, and currently multiple VHH-based drugs are on the marked. Caplacizumab (bivalent VHH targeting von Willebrand factor) was first approved by EMA in 2018 and later by the FDA in 2019 [19–21], ciltacabtagene autoleucel (CAR T-cell therapy that uses two VHHs against two different epitopes of the B-cell maturation antigen (BCMA) as targeting moieties) recently got approval of the FDA [22], envafolimab (anti-PDL1 VHH fused to the Fc domain of human IgG1) has been approved for clinical use in 2021 by the Chinese National Medical Products Administration [23] and Taisho Pharmaceutical recently filed for regulatory approval of ozoralizumab (trivalent VHH with two VHHs targeting TNF and one serum albumin for half-life extension) in Japan [24].

Multiple anti-TfR mAbs are under clinical investigation (NCT03568175 [3], NCT04251026 [9]) for drug delivery of therapeutic proteins to the CNS, and one drug recently got market approval in Japan (Izcargo(R)) [25]. Interestingly, constructs under investigation represent different TfR binding stoichiometries, namely monovalent versus bivalent binding. The role of TfR binding valency in RMT has been extensively investigated with contradictory outcomes. Some groups claim monovalent binding is crucial for transcytosis, since bivalent binding leads to lysosomal degradation [7, 8]. Another hypothesis states that this lysosomal sorting is due to high affinity binding [26–28]. As a result, a variety of TfR-binding constructs have recently been described to deliver therapeutics to the CNS, such as a bivalent VNAR [11, 12], a monovalent engineered Fc fragment [9, 10] and two scFvs fused to a mAb via a short linker to prevent bivalent binding via steric hindrance [7].

Here, we describe the discovery of an anti-human TfR (hTfR) VHH that is able to penetrate the CNS in hTfR-engineered mice using our previously described approach [15]. We also show the transport of an anti-β-secretase 1 (BACE1) antibody fused to both our anti-mTfR and hTfR VHHs to the CNS after peripheral administration in wild-type and hTfR-engineered mice respectively. The pharmacodynamic effect induced by the BACE1 inhibition of a mAb is an unambiguous proof-of-concept of the CNS-penetrating capacities of the anti-TfR VHHs. Anti-mouse TfR (mTfR) VHHs could be valuable research tools, whereas anti-hTfR VHHs have the potential to be generic tools for therapeutic protein delivery to the CNS in human patients.

## Material and methods

### VHH library generation

Targeted VHH libraries were obtained in collaboration with the VIB Nanobody Core. Two alpacas who had already been subjected to 4 bi-weekly subcutaneous (SC) immunizations with a suspension of human brain capillaries, where boosted once with 90 µg of hTfR (11020-H10H, Sino Biological), followed by another three injections of 45 µg of hTfR at two week intervals. On day 40 a blood sample of 100 ml was collected and peripheral blood lymphocytes were isolated. The VHH encoding genes were recovered and the phagemid library was cloned as previously prescribed [29]. Briefly, total RNA from peripheral blood lymphocytes was used as template for first strand cDNA synthesis with oligodT primer. This cDNA was used to amplify the VHH-encoding open reading frames by PCR, digested with PstI and NotI, and cloned into the phagemid vector pMECS. The library was transformed into electro-competent *E. coli* TG1 cells, which resulted in 10^8^ independent transformants, of which 85% contained the vector with a right insert size.

### Isolation of anti-human TfR VHHs

To select anti-hTfR VHHs, one round of in solution selection was performed with 50 nM in house biotinylated hTfR (11020-H10H, Sino Biological). Next, the library was subcloned into an expression vector (pBDS100, a modified pHEN6 vector with an OmpA signal peptide and a C-terminal 3xFlag/6xHis tag) [30]. The expression library was used to transform TG1 *E.coli* after which VHHs were expressed from single colonies. These VHHs were screened for direct binding to the biotinylated chimeric protein alpaca TfR with human apical domain (hAPI) using the AlphaScreen FLAG (M2) Detection Kit (6760613C, PerkinElmer). The hits were sequenced and clustered according to sequence homology. One representative of each sequence cluster was recloned into our neurotensin (NT) vector (pBDS100 with C-terminal NT(8-13)), expressed and purified following the protocol by Pardon et al. [29] After purification and a freeze/thaw cycle, the identity and integrity of the purified VHHs was confirmed by mass spectrometry.

### Bio-layer interferometry

Binding of the VHHs and mAbs to TfR was assessed using an Octet RED96 (Forté Bio/Molecular Devices). Briefly, streptavidin (SA) biosensor tips (18-5020, Forté Bio/Molecular Devices) were pre-wet for minimally 10 min in 1xPBS, after which they were dipped in biotinylated target protein (1 µg/ml in 1xPBS). hTfR (2474-TR, RandD) and chimeric hTfR carrying the mouse apical domain (hTfR-mAPI, produced at VIB Protein Service Facility) were biotinylated with the EZ-Link NHS-PEG4-Biotinylation Kit (ThermoFischer Scientific) according to the manufacturer instructions. Next, the tips were sequentially submerged in baseline wells (1xPBS), dissociation wells (1xPBS) to equilibrate the sensors in dissociation buffer, VHHs or monoclonal antibodies diluted in 1xPBS, and finally back into the dissociation wells for the actual dissociation. Sensograms were generated using the Forté Bio Octet RED analysis software (Forté Bio/Molecular Devices).

### Monoclonal antibodies

**Fig. 1:**
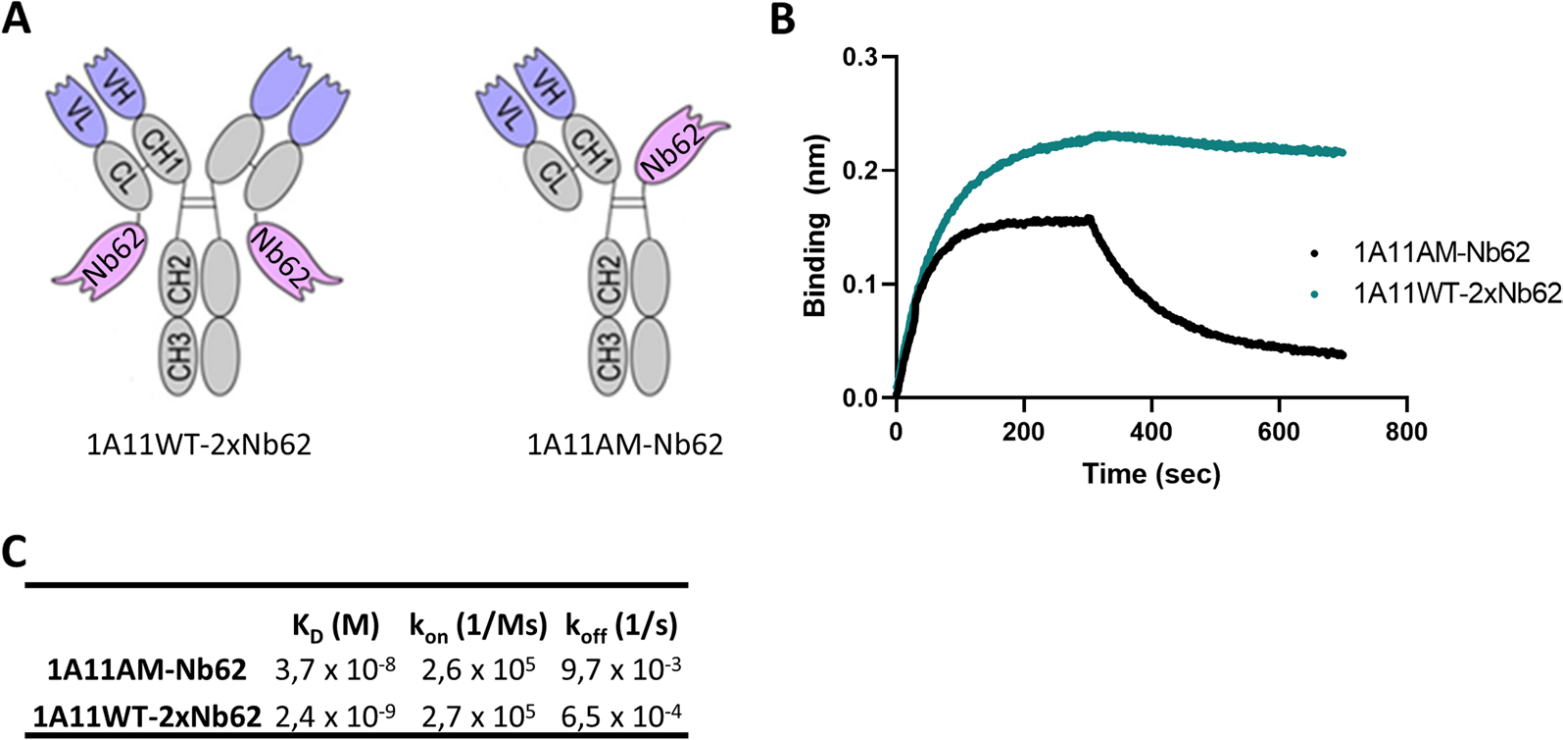
Overview and binding kinetics of anti-TfR/BACE1 antibodies. **A** The fusion of 1A11WT to two copies of Nb62 resulted in a homodimeric antibody where Nb62 was fused to the light chain of 1A11WT (left). 1A11AM-Nb62 (right) is a heterodimeric antibody where both the BACE1 inhibiting Fab fragment of 1A11AM, as well as Nb62, were each individually fused to one of the respective Fc constant domains. **B** Binding of anti-TfR/BACE1 antibodies to biotinylated mTfR immobilized on streptavidin biosensors is shown. **C** Binding kinetics were calculated and summarized

1A11WT-2xNb62 and 1A11WT were custom made by GenScript Biotech (Fig. 1A), with 1A11 being our in-house developed anti-BACE1 antibody [31]. Briefly, both the heavy chains (mouse IgG2a) and light chains (mouse kappa) was cloned into the mammalian expression vector pcDNA3.4, expressed from Expi293F™ human cells (A14527, ThermoFischer Scientific) and purified using HiTrap® MabSelect^™^ columns (GE11-0034-93, Cytiva). Its purity was estimated by densitometric analysis of the Coomassie Blue-stained SDS-PAGE gel under non-reducing conditions, and resulted in 75% (1A11WT-2xNb62). The DNA encoding for 1A11AM-Nb62 (with 1A11AM being the humanized version of 1A11WT), 1A11AM-Nb188 and 1A11AM-aGFP (with aGFP being an anti-green fluorescent protein (GFP) VHH) was synthesized by Twist Bioscience (CA, USA) and cloned in their pTwist CMV BetaGlobin WPRE Neo vector: Nb62-Fc, Nb188-Fc and aGFP-Fc (human IgG1, L234A, L235A, P329G, T350V, T366L, K392L, T394W), 1A11AM heavy chain (human IgG1, L234A, L235A, P329G, T350V, L351Y, F405A, Y407V) and 1A11AM light chain (human kappa). 1A11AM and 1A11WT bind with similar affinity to BACE1. The antibodies were expressed in Hek293F cells using the X-tremeGENE™ HP DNA Transfection Reagent (6366546001, Merck) and purified following the protocol by Nesspor et al. [32]. The purification protocol consisted of a protein A purification, followed by a purification over a CaptureSelect^™^ CH1-XL Pre-packed Column (494346205, ThermoFischer Scientific).

### Humanized TfR mice

Custom made humanized Tfrc in C57BL/6 mice (Cyagen, CA, USA) were generated by replacing the sequence encoding for amino acids 196–381 of the mouse TfR by the sequence encoding for amino acids 194–379 of the human TfR including the introns. This region is known as the apical domain of TfR. C57BL/6 embryonic stem (ES) cells were electroporated with the targeted DNA, followed by G418 selection, clonal selection and clonal amplification. Correctly targeted clones were confirmed by PCR and Southern blot analysis. Two ES cell lines were microinjected into blastocyst embryos, which were then transferred into surrogate mothers. A male chimera was bred to WT females to generate F1 heterozygous mice. Homozygous mice were generated by breeding F1 generation. The resulting chimeric mouse expresses TfR with hAPI under control of the endogenous promoter.

### Anipill® implantation

To automatically measure body temperature of socially housed mice, the Anipill® (BodyCap) system was used. Mice were injected with buprenorphine (0.05 mg/kg, SC) one hour before Anipill® implantation, followed by lidocaine (6 mg/kg, SC) as local analgesia 5 min before implantation. The mice were anesthetized with 1–2% isoflurane, respiration was monitored and temperature was maintained at 37 °C. The abdomen was opened and the Anipill® was implanted. Next, the muscle layer was sutured with resorbable sutures and the skin was closed with surgical staples. Then, 500 µl of saline was injected SC and the animals were allowed to recover under a heating lamp, followed by an additional injection of buprenorphine (0.1 mg/kg, SC) 6 h later. The Anipill® implantation was performed at least 1 week prior to any follow up experiment. Body temperature was monitored every 15 min using the Anipill® system.

### Intravenous injections

For intravenous injections of VHH-NT fusions, the mice were put in a restrainer and the tail was heated in warm water between 42 and 48 °C. For intravenous injections of mAbs, the mice were put in a heating chamber at 40 °C Celsius for 10 min. Then, the mice were put in a restrainer and mAbs were injected in the tail vein at volumes between 100 and 180 µl. All animal experiments were conducted according to protocols approved by the local Ethical Committee of Laboratory Animals of the KU Leuven (governmental license LA1210579, ECD Project Number 213/2020) following governmental and EU guidelines.

### Plasma/brain sampling

Mice were euthanized with a Dolethal overdose (150–200 mg/kg) injected intraperitoneally. To harvest plasma, blood was collected with a prefilled heparin syringe via cardiac puncture. Next, blood samples were spun at 2000 g for 10 min and plasma was collected. Brains were harvested after transcardial perfusion with heparinized PBS.

### *Aβ*_*1–40*_* detection using MSD ELISA*

Mouse Aβ_1–40_ samples from brain and plasma were prepared according to Serneels et al. [33] Briefly, for brains, a hemisphere was homogenized in buffer containing 20 mM Tris, 250 mM sucrose, 0.5 mM EDTA, 0.5 mM EGTA (pH 7.4 HCl) supplemented with cOmplete^™^ protease inhibitor cocktail (Roche) and PhosSTOP^™^ (Sigma) using a bead mill. Next, soluble Aβ_1–40_ was extracted by 0.4% diethylamine treatment for 30 min at 4 °C, high speed centrifugation (100 000 g, 1 h, 4 °C) and neutralization with 0.5 M Tris–HCl (pH 6,8). Aβ_1–40_ levels were quantified by ELISA using Meso Scale Discovery (MSD) 96-well plates and antibodies provided by Janssen Pharmaceutica. MAb JRFcAβ40/28, which recognizes the C terminus of Aβ_1–40_, was used as a capture antibody and JRF/rAβ/2 labeled with sulfoTAG as the detection antibody.

### huIgG detection using MSD ELISA

Brain and plasma samples were prepared according to Kariolis et al. [10] Briefly, for brains, a hemisphere was homogenized with a bead homogenizer 10 × by tissue weight of 1% NP-40 in PBS (pH 7.4) supplemented with cOmplete™ protease inhibitor cocktail (Roche). The homogenate was incubated for 60 min at 4 °C before centrifugation (16000 g, 10 min, 4 °C). The supernatant was collected for pharmacokinetic analysis.

Antibody concentrations in plasma and brain samples were determined by using a commercial human IgG assay (MSD human/NHP IgG kit, #K150JLD) following the manufacturer's instructions. Briefly, plates were blocked for 30 min with MSD blocker A and washed with PBS-T (0.05% Tween 20). All plasma samples of day 1 and 3 were diluted 1:1000, plasma samples of day 7 containing a TfR binding construct were diluted 1:100 and the plasma samples of day 7 containing a GFP binding construct were diluted 1:1000. Brain homogenates were diluted 1:10. The brain and plasma calibration curves (49 pg/mL-200 000 pg/mL) were determined as a duplicate in their corresponding matrix. The data were analyzed using a 4-parameter, logistic curve fitting model (sigmoidal dose–response) with a 1/Y^2^ weighting function.

### Western blot

One brain hemisphere from wild-type (WT), heterozygous (Het) or hAPI knock-in (KI) mice was homogenized with a bead homogenizer 3 × in RIPA buffer (Sigma) supplemented with cOmplete EDTA-free^™^ protease inhibitor cocktail (Roche) and phosSTOP (Roche). Samples were then centrifuged at 17,000 g for 15 min at 4 °C and supernatants were collected. Protein concentration was determined with the BCA Kit (ThermoFischer Scientific). An amount of 15 µg of protein lysate was mixed with Pierce Lane Marker Reducing Sample Buffer (ThermoFischer Scientific) and heated during 5 min at 95 °C. Samples were resolved in a NuPAGE^™^ 10% Bis–Tris gel with NuPAGE^™^ MOPS SDS Running Buffer (ThermoFischer Scientific) and then transferred to a nitrocellulose membrane (Millipore) in a wet tank transfer system (ThermoFischer Scientific) at 28 mA during 1 h and 30 min. The membranes were then blocked with 5% milk and 5% bovine serum albumin (BSA) in TBST (10 mM Tris–HCl (pH 7.5), 150 mM NaCl and 1% Tween-20) for 1 h at room temperature (RT). Membranes were incubated with the following primary antibodies diluted in TBST containing 1% BSA: TfRc (1:1000; overnight at 4 °C; Invitrogen, 13-6890) and β-actin (1:5000; 20 min at RT; Sigma, A5441). Membranes were washed with TBST and incubated at RT during 1 h with goat anti-mouse HRP-conjugated immunoglobulin (DAKO; P0447) diluted 1:5000 in TBST containing 5% milk. Finally, membranes were developed with Western Lightning Plus Chemiluminescent Substrate (PerkinElmer), scanned using a LAS4000 Biomolecular imager (GE Healthcare) and analysed with ImageQuant TL version 7.0 software (GE Healthcare).

## Results

### Engineering fusions of anti-BACE1 antibody 1A11 with anti-mouse TfR Nb62

Previously we discovered Nb62, an anti-mTfR VHH that is able to deliver NT to the CNS. The next step was to assess whether Nb62 could also deliver larger proteins to the brain. For this purpose, we utilized the BACE1-inhibiting antibody 1A11 [31]. BACE1 is an enzyme involved in the production of amyloid-beta (Aβ) peptides in the periphery and brain [34], and has been used as a readout for brain penetration of engineered anti-TfR monoclonal antibodies [10, 13, 35]. In order to assess whether Nb62 is able to reach the brain in a bivalent format, we genetically fused two copies of Nb62 by a flexible glycine-serine (GGGGS)_3_ linker. This construct was fused to NT(8-13) and peripherally administered to WT mice. As can be seen in Additional file 1: Fig. S1, the bivalent version of Nb62 had a significantly decreased brain uptake compared to monovalent Nb62, indicated by the lack of hypothermia. Therefore we decided to target the TfR in a monovalent manner and we fused Nb62 to 1A11 in two ways, which are represented in Fig. 1A. The first construct (1A11WT-2xNb62) consisted of a homodimer where two copies of Nb62 are fused to the light chains of 1A11 via a short linker. This approach was based on published data by Hultqvist et al. [7], where they showed monovalent TfR targeting of an anti-TfR scFv fused in the same way to an anti-Aβ antibody. The second construct (1A11AM-Nb62) was a heterodimeric antibody where the 1A11 Fab and Nb62 were individually fused to a subunit of the Fc domain, based on a paper by Nesspor et al*. *[32].

Binding of both constructs to TfR was confirmed by bio-layer interferometry (BLI) using the Octet system (Fig. 1B). However, the binding profiles of both constructs resulted to be very different compared to each other. Whereas 1A11AM-Nb62 showed a comparable sensogram to monovalent Nb62, 1A11WT-2xNb62 displayed binding with minimal dissociation, suggesting bivalent binding despite the fact that we aimed to target TfR monovalently. The mTfR-binding affinities are shown in Fig. 1C, where it can be seen that 1A11WT-2xNb62 had a 15-fold higher apparent affinity (*K*_D_ = 2.4 × 10^–9^ M) compared to 1A11AM-Nb62 (*K*_D_ = 3.7 × 10^–8^ M). This difference was mainly driven by the dissociation rate (k_off_), as calculated from the sensogram in Fig. 1B.

### *Nb62 fused to anti-BACE1 1A11AM antibody is able to reduce Aβ*_*1-40*_* levels in the brain*

**Fig. 2:**
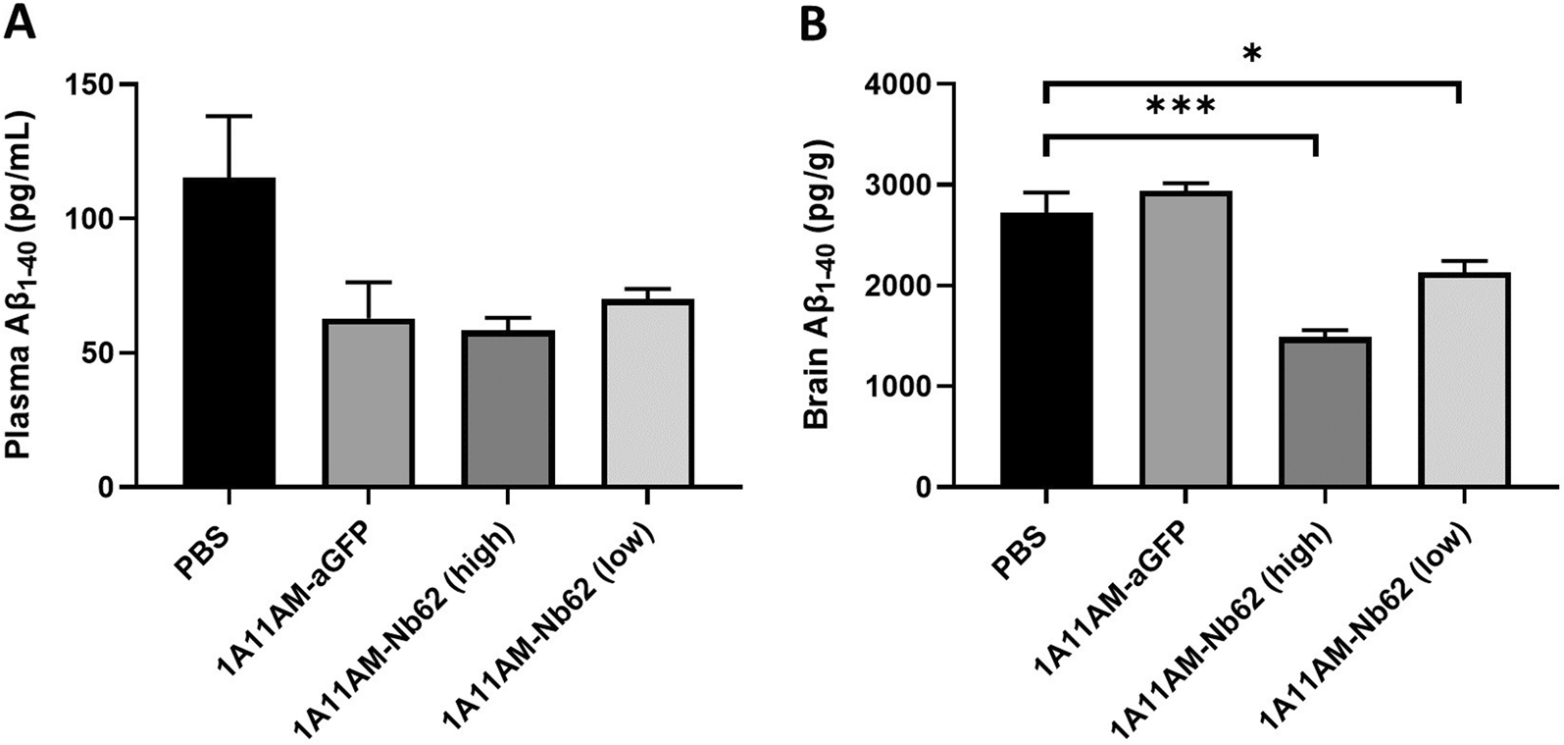
Effect on Aβ_1–40_ levels after systemic dosing with anti-mouse TfR/BACE1 antibody. Quantification of Aβ_1–40_ concentrations in C57BL/6 J mouse plasma **A** and brain **B** 24 h after an intravenous injection of PBS, 1A11AM-aGFP (167 nmol/kg) or 1A11-Nb62 (high dose: 167 nmol/kg; low dose: 16.7 nmol/kg). Bar graphs represent mean ± SEM (n = 3 per group). Statistical test: one-way ANOVA with Dunett’s multiple comparisons test compared to PBS injected mice (*p < 0.05, ***p < 0.001)

Given the avid binding of 1A11WT-2xNb62 to TfR (Fig. 1B) and the reduction in brain penetration of bivalent Nb62 fused to NT (8-13) (Additional file 1: Fig. S1), we decided to select 1A11AM-Nb62 for further in vivo validation. As a negative control, an anti-GFP VHH was fused to 1A11AM in a similar manner. Both constructs were intravenously injected in C57BL/6 WT mice, and after 24 h both plasma and brain Aβ_1–40_ levels were determined. These levels were compared to those from PBS injected mice and are displayed in Fig. 2. As can be seen in panel A, both 1A11AM-fused constructs reduced plasma Aβ_1–40_ levels, indicating peripheral BACE1 inhibition. Centrally however, only 1A11AM-Nb62 was able to reduce Aβ_1–40_ levels both at a high and low dose (Fig. 2B). This shows that Nb62 was able to deliver 1A11AM to the brain, whereas the anti-GFP VHH was not.

### Generation of a human-specific TfR VHH that is able to shuttle from the periphery into the brain

**Fig. 3:**
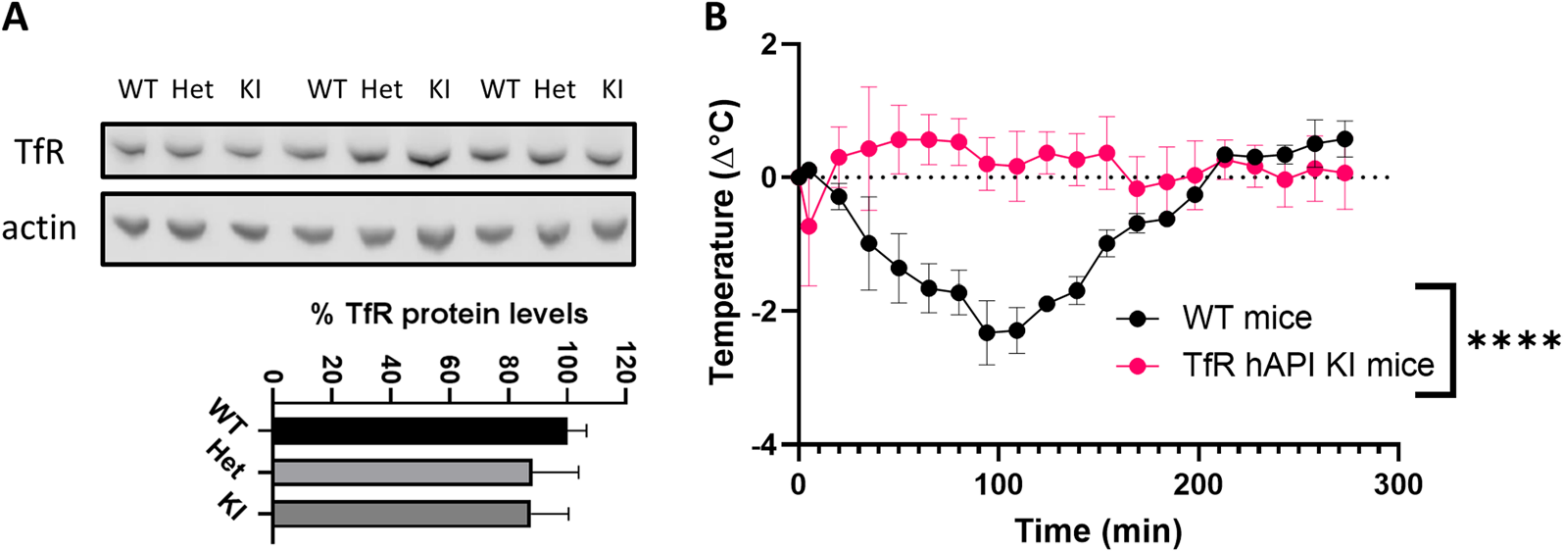
Validation of TfR hAPI KI mice. **A** Top: TfR protein levels in brain lysates of wild-type (WT), heterozygous (Het) and hAPI knock-in (KI) mice. Actin is used as a loading control. Uncropped western blot can be found in Additional file 2: Fig. S2. Bottom: Quantification of TfR protein levels normalized to WT levels. Bar graphs represent mean ± SEM (n = 3 for KI; n = 4 for WT and Het). **B** C57BL/6 WT and TfR hAPI KI mice body temperature measurements after 250 nmol/kg intravenous injections of Nb62 fused to NT(8-13). Bar graphs represent mean ± SEM (n = 3 per group). Statistical test: two-way ANOVA with repeated measures and Sidak’s multiple comparisons test. (significant time*genotype interaction effect ****p < 0.0001)

As Nb62, like many other TfR-targeting brain-penetrating antibodies [36], is not cross-reactive to hTfR, we decided to generate anti-hTfR VHHs. To be able to validate these for their brain-penetrating capabilities, a chimeric mouse model was generated, where the apical domain of the mTfR, which is where all reported TfR and brain-penetrating antibodies bind to, was replaced by the human sequence (hAPI KI mice). To validate the model, the TfR expression in the brain was analyzed by western blot and compared to C57BL/6 WT mice. The representative blot is displayed in Fig. 3A along with the quantification of the TfR levels normalized to the WT levels. No significant difference in TfR expression levels was observed between WT, heterozygous and hAPI KI mice. In order to validate the successful knockout of the mouse apical domain, the mouse-specific apical domain binder Nb62 fused to neurotensin was intravenously injected and body temperature was recorded (Fig. 3B). Whereas Nb62 elicited a decrease in body temperature in C57BL/6 WT mice, this effect was lost in the hAPI KI mice, indicating knockout of the mouse apical domain (area under the curve (AUC) respectively 274 ± 24 (WT mice) and 71 ± 35 (TfR hAPI KI mice) °C x min, ****p < 0.0001 (unpaired t test with Welch’s correction)).

**Fig. 4:**
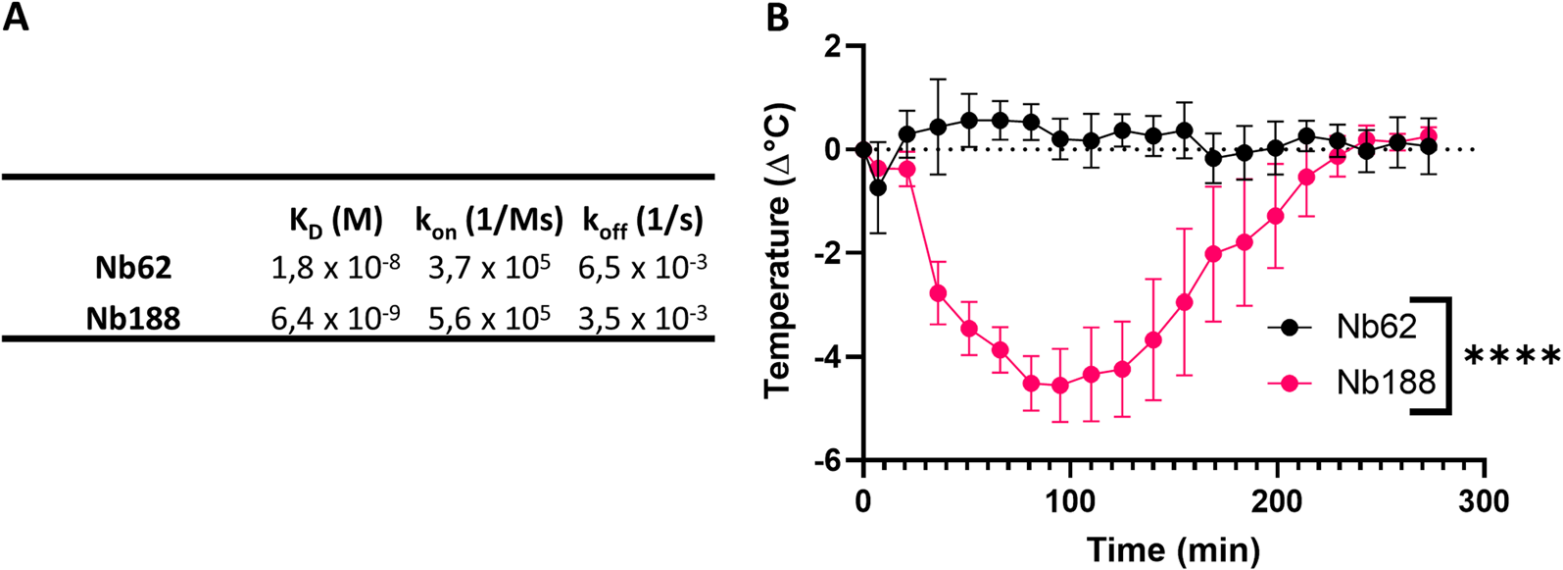
Characterization of anti-hTfR Nb188 in TfR binding and shuttling over the BBB. **A** Binding kinetics of Nb62 and Nb188 to immobilized mTfR and hTfR, respectively, were determined by BLI using the Octet system. **B** TfR hAPI KI mice body temperature measurements after 250 nmol/kg intravenous injections of the indicated VHH fused to NT(8-13). Bar graphs represent mean ± SEM (n = 3 per group). Statistical test: two-way ANOVA with repeated measures and Sidak’s multiple comparisons test. (significant time*treatment interaction effect ****p < 0.0001)

In order to generate brain-penetrating anti-hTfR VHHs, we performed in vitro phage display on the hTfR using a VHH-displaying phage library originating from camelids immunized with hTfR. Single clones from the output library were screened for their binding to the human and mouse TfR. Unfortunately, none of the anti-hTfR were cross-reactive to mTfR. Next, the hits were screened for apical domain binding by using recombinant alpaca TfR with human apical domain. One hit, Nb188, was purified and binding to the hTfR was confirmed by BLI using the Octet system (Fig. 4A). Its binding affinity for hTfR (*K*_D_ = 6.4 × 10^–9^ M) was 35-fold higher compared to that of Nb62 (*K*_D_ = 1.8 × 10^–8^ M) to mTfR. Even though it is claimed that a high TfR-binding affinity reduces brain uptake via RMT in mice [28], Nb188 fused to NT was peripherally administered to the hAPI KI mice in order to assess its brain penetration. As can be seen in Fig. 4B, Nb188 was able to reach the brain, indicated by the drop in body temperature (AUC: 609 ± 72 (NB188) and 71 ± 35 (NB62) °C x min, ****p < 0.0001 (unpaired t test with Welch’s correction)).

### *Human-specific TfR VHH fused to BACE1 1A11AM antibody reduces Aβ*_*1-40*_* levels in the brain*

**Fig. 5:**
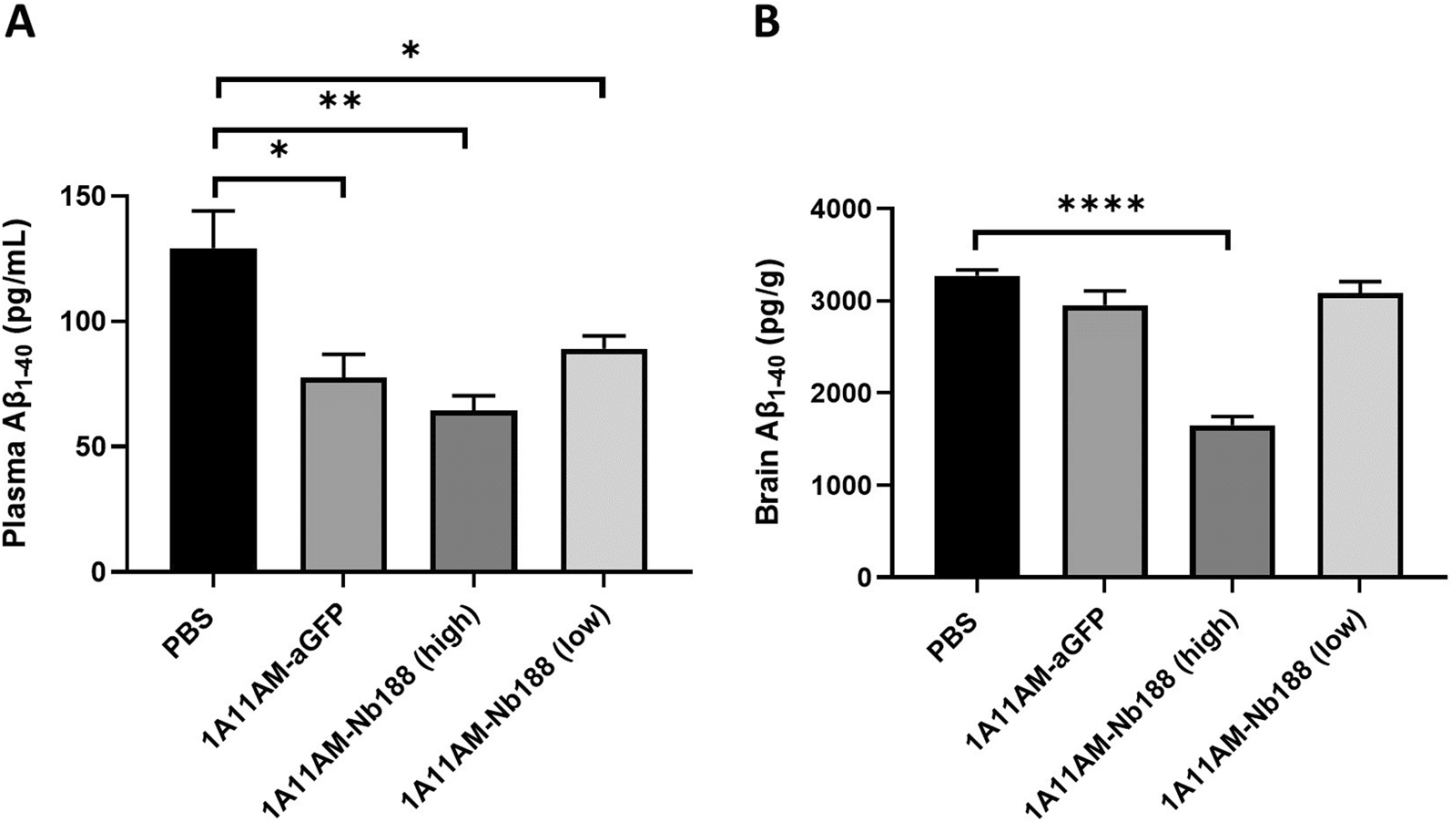
Effect on Aβ_1–40_ levels after systemic dosing with anti-human TfR/BACE1 antibody. Quantification of Aβ_1–40_ concentrations in TfR hAPI KI mouse plasma **A** and brain **B** 24 h after an intravenous injection of PBS, 1A11AM-aGFP (167 nmol/kg) or 1A11AM-Nb188 (high dose: 167 nmol/kg; low dose: 16.7 nmol/kg). Bar graphs represent mean ± SEM (n = 3 per group). Statistical test: one-way ANOVA with Dunnet’s multiple comparisons test compared to PBS injected mice. (*p < 0.05, **p < 0.01, ****p < 0.0001)

Next, similar to Nb62 (Fig. 2), we assessed how well Nb188 can shuttle an anti-BACE1 Fab (1A11AM-Nb188) into the brain. The same negative control (1A11AM-aGFP) was used. Both constructs were intravenously injected in hAPI KI mice, and plasma and brain Aβ_1–40_ levels were determined 24 h post injection. These levels were compared to those from PBS injected mice and are displayed in Fig. 5. Again, both constructs were able to inhibit peripheral BACE1, as shown by the decrease of plasma Aβ_1–40_ levels (Fig. 5A), whereas only the high dose of anti-TfR targeted construct (1A11AM-Nb188) was able to reduce Aβ_1–40_ levels in the brain (Fig. 5B). This validates Nb188 for the delivery of large molecules to the CNS.

### PK/PD study of mouse and human-specific TfR/BACE1 antibodies

**Fig. 6:**
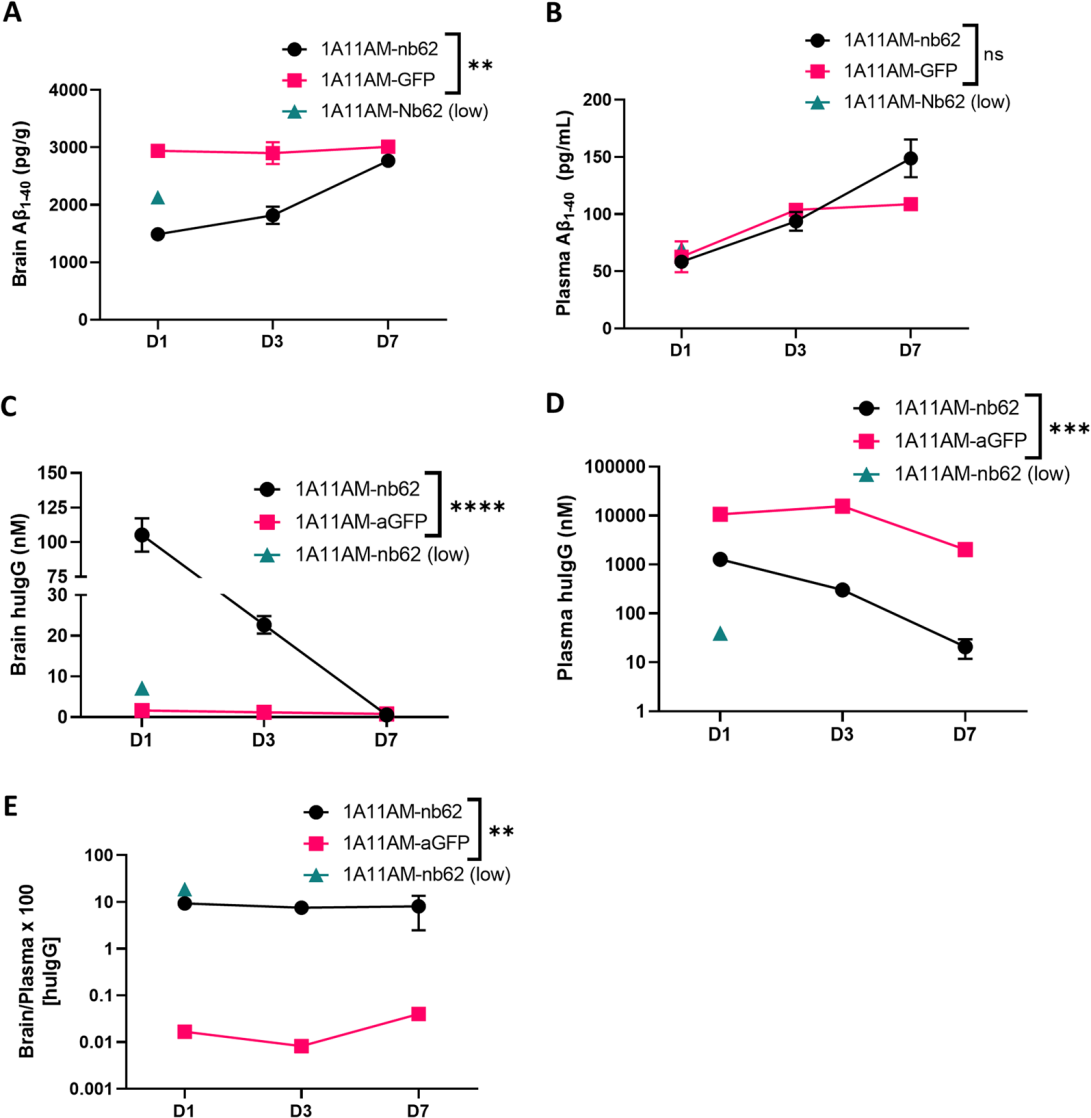
PK/PD with anti-mouse TfR/BACE1 antibody. Quantification of Aβ_1–40_ **A, B** and huIgG concentrations **C**, **D** in C57BL/6 J WT mouse brains **A**, **C** and plasma **B**, **D** at day **D **1, 3 and 7 after a single intravenous injection of 1A11AM-aGFP or 1A11AM-Nb62 (167 nmol/kg; low dose: 16.7 nmol/kg). Bar graphs represent mean ± SEM (n = 3–5 per group). Statistical test: two-way ANOVA with Sidak’s multiple comparisons test. **A** and **B** Significant treatment*day interaction effect for brain (**p = 0.0014), but not for plasma (ns = 0.0529). **C** and **D** Significant treatment*day interaction effect for both brain (****p < 0.0001) and plasma (***p < 0.0002). E) HuIgG brain over plasma ratio. Significant overall treatment effect (**p = 0.0027). Significant overall treatment effect for **A**, **C**, **D**, **E** and significant effect over time for **A**, **B**, **C**, **D** not shown

**Fig. 7:**
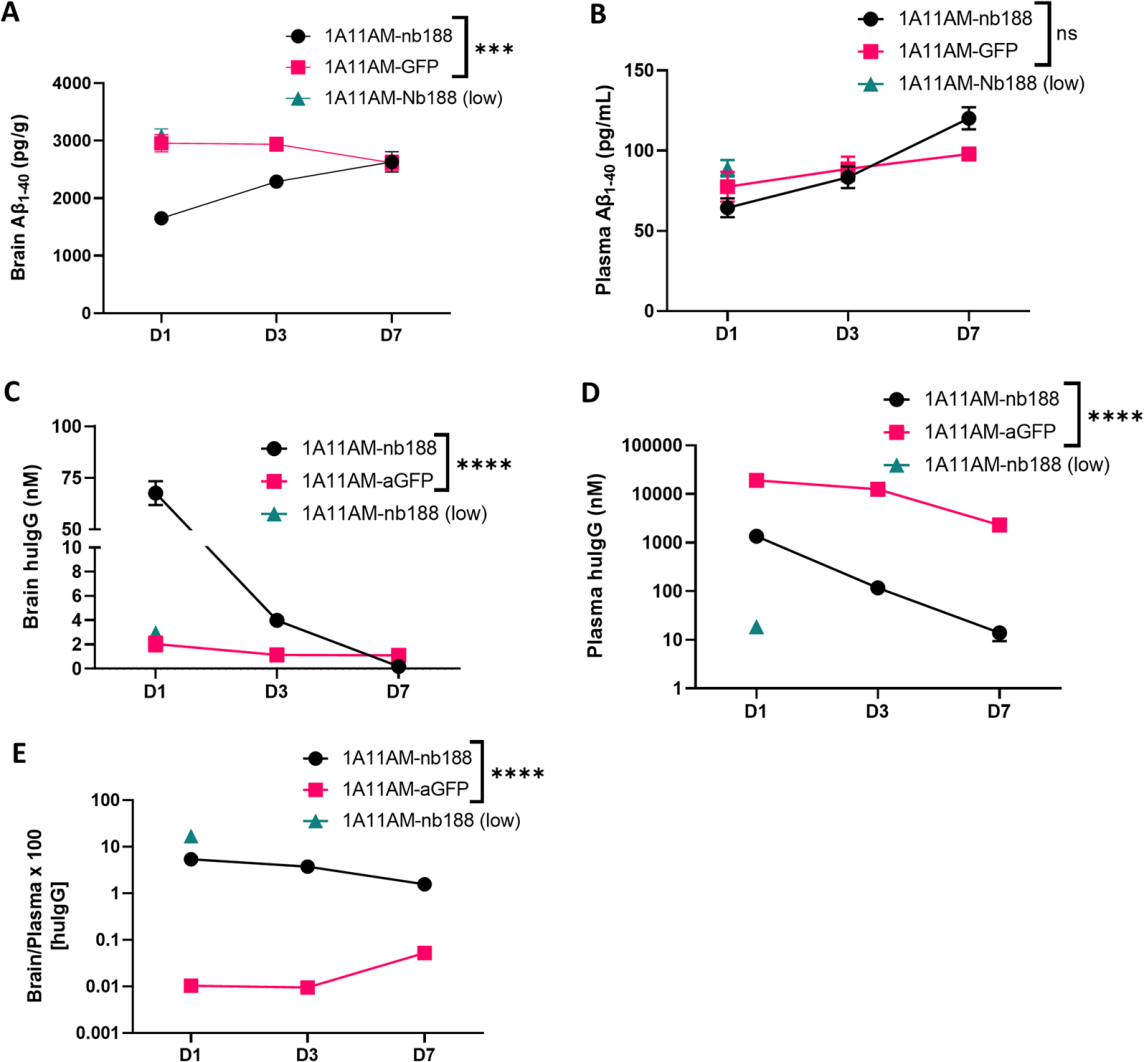
PK/PD with anti-human TfR/BACE1 antibody. Quantification of Aβ_1–40_ **A, B** and huIgG concentrations **C**, **D** in hAPI KI mouse brains **A**, **C** and plasma **B**, **D** at day **D** 1, 3 and 7 after a single intravenous injection of 1A11AM-aGFP or 1A11AM-Nb188 (167 nmol/kg; low dose: 16.7 nmol/kg). Bar graphs represent mean ± SEM (n = 3–5 per group). Statistical test: two-way ANOVA with Sidak’s multiple comparisons test. **A** and **B** Significant treatment*day interaction effect for brain (***p = 0.0006), but not for plasma (ns = 0.0637). **C** and **D**. Significant treatment*day interaction effect for both brain and plasma (****p < 0.0001). **E** HuIgG brain over plasma ratio. Significant overall treatment effect (*p = 0.0120). Significant overall treatment effect for **A**, **C**, **D**, **E** and significant effect over time for **B**, **C**, **D**, **E** not shown

Next, the pharmacokinetic and pharmacodynamic (PK/PD) profiles for 1A11AM-Nb62 and 1A11AM-Nb188 were determined (respectively Figs. 6, 7). Hereto, the antibody levels (PK) as well as the Aβ_1-40_ levels (PD) were determined in brain and plasma at day 1, 3 and 7 post injection. The plasma levels of the constructs with an anti-TfR VHH were significantly lower than the levels of isotype (anti-GFP) control containing antibodies, indicating target mediated clearance of the TfR targeted constructs (Fig. 6D (p = 0.0005 (D1) and p < 0.0001 (D3)) and Fig. 7D (p < 0.0001 (D1) and p < 0.0001 (D3))). However, despite the faster clearance, the concentration in the brain was significantly higher for both constructs at day 1 and for1A11AM-Nb62 at day 3. At day 7 they both returned to the level of the isotype control (Fig. 6C (p < 0.0001 (D1) and p = 0.0047 (D3)) and Fig. 7C (p < 0.0001 (D1) and p = 0.5652 (D3))). Of note, the reported concentrations represent the total brain concentration of the respective constructs, including the constructs trapped in the capillaries after perfusion as we opted to not deplete the capillaries. In accordance to the plasma levels, the Aβ_1-40_ levels in the brain were also significantly decreased at day 1 and 3, but not at day 7 for both constructs (Fig. 6A (p < 0.0001 (D1) and p < 0.0001 (D3)) and Fig. 7A (p < 0.0001 (D1) and p = 0.0041(D3))). Peripherally, no difference in Aβ_1-40_ levels could be observed for any of the constructs (Figs. 6B and 7B).

## Discussion

Currently, different receptors are being proposed to shuttle drug cargos over the BBB, e.g. TfR, InsR, IGF1R and CD98hc [3, 4, 13, 14]. At present, anti-IGF1R and CD98hc binding moieties to shuttle cargos over the BBB are still in preclinical development, while anti-InsR antibodies are in mid-clinical development and anti-TfR antibodies are currently approved in Japan [4, 25]. Unfortunately, so far no studies have been done to directly compare the transport efficiencies of the different receptors, and the different affinity binders which exists for each receptors. Generally spoken, TfR has been studied most extensively as BBB shuttling receptor, by multiple academic and industry groups [5–12]. Transport is robust and initial safety issues seem to have been overcome [35]. One of the potential downsides of TfR as BBB shuttling receptor is its ubiquitous expression [37]. This may lead to unintended accumulation of the drug containing the anti-TfR binding moiety in TfR expressing tissues, which may lead to unwanted side effects. Also, this leads to target mediated clearance and hence a shorter peripheral half-life, as is also shown in our study [10, 28]. In a clinical setting, more frequent dosing might be required to keep levels high enough to elicit therapeutic effect. Interestingly, this fast clearance was not observed for the anti-TfR VNAR, hinting towards a distinct epitope for this VNAR which is only available at the BBB [12]. Also, most anti-TfR binding affinity binders are not cross reactive between human and mouse TfR which could complicate preclinical studies. However, this last issue has been overcome by generating human TfR knock in mice, as also demonstrated by us in this paper. Also for InsR, the BBB-shuttling antibody lacks cross reactivity, while for CD98hc and IGF1R this seems to be less of an issue [5, 13, 14, 38].

Previously, we described the discovery of an anti-mouse TfR VHH, Nb62, and its potential to deliver NT to the CNS [15]. However, the hypothermic effect of NT is mediated specifically by hypothalamic neurons [39], which does not necessarily mean that Nb62 is able to target the entire brain. Moreover, the dose at which the hypothermic effect is observed, might be therapeutically irrelevant. Therefore, in order to validate Nb62 by a more pharmacologically relevant approach, we fused it to a therapeutically relevant protein, i.e. an antibody. As a readout for CNS-targeting of Nb62, we relied on BACE1 inhibition. Besides plasma Aβ_1–40_ levels, BACE1-inhibiting antibodies have been shown to decrease also brain Aβ_1–40_ levels when administered at a high dose [40], and at lower doses when engineered to simultaneously bind TfR for enhanced CNS delivery [10, 35]. The TfR has been extensively investigated to deliver therapeutic proteins to the CNS, with one of the main outstanding questions being the impact of anti-TfR-binding valency on brain penetration efficiency [7, 8, 27, 28]. Currently, multiple clinical trials are ongoing that utilize TfR mediated transport of therapeutic proteins to the CNS, respectively with monovalent and bivalent TfR binding [3, 9]. Our results showed that bivalent targeting of TfR by Nb62 leads to a reduction in brain penetration (Additional file 1: Fig. S1), which is in accordance with literature, where it is shown that bivalent targeting of the TfR can lead to lysosomal sorting and degradation [8]. Therefore, we explored two approaches to engineer the anti-BACE1 antibody 1A11 with Nb62 in order to obtain monovalent TfR binding (Fig. 1A). One construct (1A11WT-2xNb62) was a homodimer generated in a similar way as two anti-TfR scFvs fused to an anti-Aβ antibody via a short linker that should sterically prevent simultaneous binding of both VHHs to TfRs (no avidity) [7]. The other antibody construct (1A11AM-Nb62) was generated according to Nesspor et al*. *[32], and consisted of the 1A11AM Fab and one copy of Nb62 both respectively fused to one subunit of the Fc domain. BLI analysis indicated very distinct binding profiles of both bispecific antibodies to TfR (Fig. 1B). Whereas 1A11AM-Nb62 showed an association and dissociation curve with an affinity of 37 nM, 1A11WT-2xNb62 showed minimal dissociation, translating to a stronger apparent binding affinity of 2.4 nM (Fig. 1C). This indicates that 1A11WT-2xNb62 can avidly bind to TfR, despite the short linker that previously prevented an anti-TfR scFv from avid binding [7]. As bivalent binding of NB62 prevented its BBB crossing in the NT model (Additional file 1: Fig. S1), only the bispecific antibody binding monovalently to TfR (1A11AM-Nb62) was further validated in vivo. This bispecific antibody was intravenously injected followed by the determination of Aβ_1-40_ levels in plasma and brain. As a negative control, Nb62 was replaced by an irrelevant VHH binding to GFP. Both constructs showed a clear tendency towards peripheral Aβ_1-40_ levels (Fig. 2A) compared to PBS injected mice, however due to the variability in the PBS injected group this difference was not statistically significant. In contrast, only 1A11AM-Nb62 was able to inhibit BACE1 centrally at both high and low dose (Fig. 2B) by 45 and 22%, respectively. This shows that Nb62 is able to deliver an anti-BACE1 mAb to the CNS at a pharmacological relevant concentration. The extent of BACE1 inhibition (45%) at the highest dose was slightly larger compared to the published percent inhibition of Genentech’s anti-mTfR/BACE1 antibody at an equimolar dose (∼ 36%) [40]. This might indicate that Nb62 is more efficient in the delivery of an anti-BACE1 inhibiting antibody to the CNS, although this remains speculative without a side-by-side comparison. One hint that Nb62 is a highly efficient transport tool, is the significant decrease in brain Aβ_1–40_ levels following a tenfold lower dose injection of 1A11AM-Nb62 (Fig. 2B). To our knowledge, this is the lowest peripheral dose of a BACE1-inhibiting antibody reported in literature that is able to exert a pharmacodynamic effect.

Unfortunately, Nb62, like many other brain-penetrating anti-TfR antibodies [10, 27, 28, 35, 41], is not cross-reactive between mouse and human TfR [15]. Therefore we initiated the discovery of anti-hTfR VHHs that are able to deliver therapeutics to the CNS. Since all reported brain-penetrating antibodies up to date bind the apical domain of the receptor, we focused on this epitope. To allow in vivo validation, we generated a mouse model where the apical domain of the TfR was replaced by the human sequence. The TfR protein levels did not differ in heterozygous and full KI mice in comparison to WT mice (Fig. 3A), showing that the replacement of the apical domain did not alter TfR expression. Next, Nb62, which crosses the BBB in WT mice by binding to the mouse TfR apical domain but which is not cross reactive to human TfR, was not able to deliver NT(8-13) to the CNS, as indicated by the absence of the hypothermic effect. Therefore, we can conclude that the mouse apical domain was successfully removed. Next, an anti-hTfR VHH-displaying phage library was generated. By performing phage selections on the hTfR and screenings on a chimeric alpaca TfR with human apical domain, we were able to select Nb188 as a potential VHH of interest. Cross-reactivity to the mTfR was explored, but unfortunately Nb188 was not cross-reactive. Next, its binding affinity was determined and compared to that of Nb62 binding to the apical domain of mTfR by BLI using the Octet system. As can be seen in Fig. 4A, Nb188 has a 35-fold stronger binding to the hTfR compared to Nb62 to a mTfR. According to literature a high binding affinity is undesirable for CNS penetration [26, 28], whereas others describe efficient brain penetration using high affinity TfR binding [12]. Nevertheless, Nb188 was fused to NT (8-13) and intravenously injected in the hAPI KI mice. From Fig. 4B it can be seen that Nb188 was able to reach the brain, indicated by the drop in body temperature.

Similar to Nb62, the next step was to validate Nb188 for the delivery of a mAb to the CNS. Here, we fused Nb188 monovalently to 1A11AM in the same way as described above, and administered it to the hAPI KI mice. The same controls (PBS and 1A11AM-aGFP) were used to determine baseline BACE1 activity and peripheral BACE1 inhibition. Both 1A11AM constructs were able to decrease plasma Aβ_1–40_ levels, whereas only the high dose (167 nmol/kg) of TfR-targeted antibody was able to reduce brain Aβ_1–40_ levels (Fig. 5). These results validate Nb188 for the delivery of therapeutically relevant concentrations of antibody to the CNS. The extent of central BACE1 inhibition (50%) was similar to 1A11AM-Nb62 (45%, Fig. 2B), which is close to the speculated maximum in vivo Aβ_1–40_ reduction following allosteric BACE1 inhibition [31, 35, 40]. Nb188 however is less efficient in delivering 1A11AM to the CNS compared to Nb62, since it was not able to inhibit BACE1 after a systemic dose of 16.7 nmol/kg. This is also reflected by the brain PK data, where it can be observed that 1A11AM-Nb188 had a faster clearance from the brain (Fig. 7C) compared to 1A11AM-Nb62 (Fig. 6C), especially when considering the concentrations on day 3 post injection (94.1% ± 1.5 vs*.* 78,4 ± 4.1% reduction respectively between day 1 and 3). It is highly likely that the faster clearance is target mediated, especially given the fact that the major difference between both constructs is the stronger affinity of Nb188 compared to Nb62 for TfR. Nevertheless, a significant decrease in brain Aβ_1–40_ levels for both constructs was still observed three days post peripheral administration. In literature, anti-hTfR/BACE1 constructs lead to ∼ 30 and 57% BACE1 inhibition 24 h post injection in hTfR KI and hAPI KI mice, respectively [10, 35]. These constructs were administered at 333 nmol/kg, which is twice the dose of 1A11AM-Nb188 (167 nmol/kg) used in our study. The reason for us to select this dose was that Atwal et al. [40] showed that their non-TfR targeted BACE1 inhibiting antibody was able to reduce brain Aβ_1–40_ levels at a dose of 333 nmol/kg, but not at 167 nmol/kg. This difference in administered dose and the fact that another anti-BACE1 inhibiting antibody (which might have a different potency) is used, makes it impossible to make a direct comparison in terms of CNS-penetrating capacities based on PD data alone.

## Conclusion

In conclusion, we discovered an anti-hTfR VHH that is able to penetrate the CNS using our previously described approach [15]. We also validated this VHH (Nb188) together with our previously discovered anti-mTfR VHH (Nb62) for the therapeutic delivery of an anti-BACE1 antibody. In general, our constructs appear to be very similar in terms of CNS penetration compared to previously published anti-TfR affinity binders. However, the CNS penetration efficiency of Nb188 could potentially be even further improved by optimizing its affinity for the hTfR. Overall, we discovered two highly interesting anti-TfR VHHs that can respectively be a valuable research tool in mice and a potentially therapeutically relevant moiety for human patients to deliver biologicals to the CNS.

## Supplementary Information

**Additional file 1: Figure S1.** Bivalent Nb62 fails to shuttle NT(8-13) into the brain. Mouse body temperature measurements are shown after 250 nmol/kg intravenous injections of the indicated VHH fused to NT(8-13). Bar graphs represent mean ± SEM (n = 3 per group). Statistical test: two-way ANOVA with repeated measures and Sidak’s multiple comparisons test. (significant time*treatment interaction effect ****p<0.0001).

**Additional file 2: Figure S2.** uncropped Western blot images from figure 3A. Blot was cut horizontally prior to staining to allow simultaneous staining for anti-TfR (upper panel) and B-Action (lower panel).

## Data Availability

The datasets used and/or analyzed in the current study are available from the corresponding authors upon reasonable request.
