# Supplementary figures and images for: VHHs as tools for therapeutic protein delivery to the central nervous system
Source: Fluids Barriers CNS. 2022 Oct 3;19:79. doi: 10.1186/s12987-022-00374-4 (PMC9531356; doi:10.1186/s12987-022-00374-4)

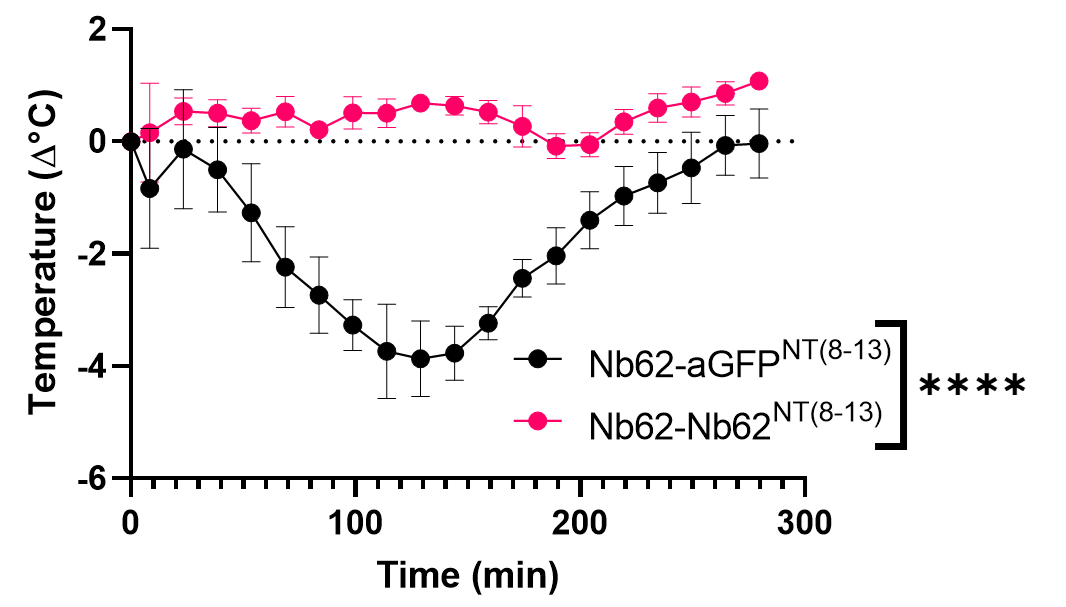

Supplement: Supplementary file 1 — Additional file 1: Figure S1. Bivalent Nb62 fails to shuttle NT(8-13) into the brain. Mouse body temperature measurements are shown after 250 nmol/kg intravenous injections of the indicated VHH fused to NT(8-13). Bar graphs represent mean ± SEM (n = 3 per group). Statistical test: two-way ANOVA with repeated measures and Sidak’s multiple comparisons test. (significant time*treatment interaction effect ****p<0.0001). [file 12987_2022_374_MOESM1_ESM.png]

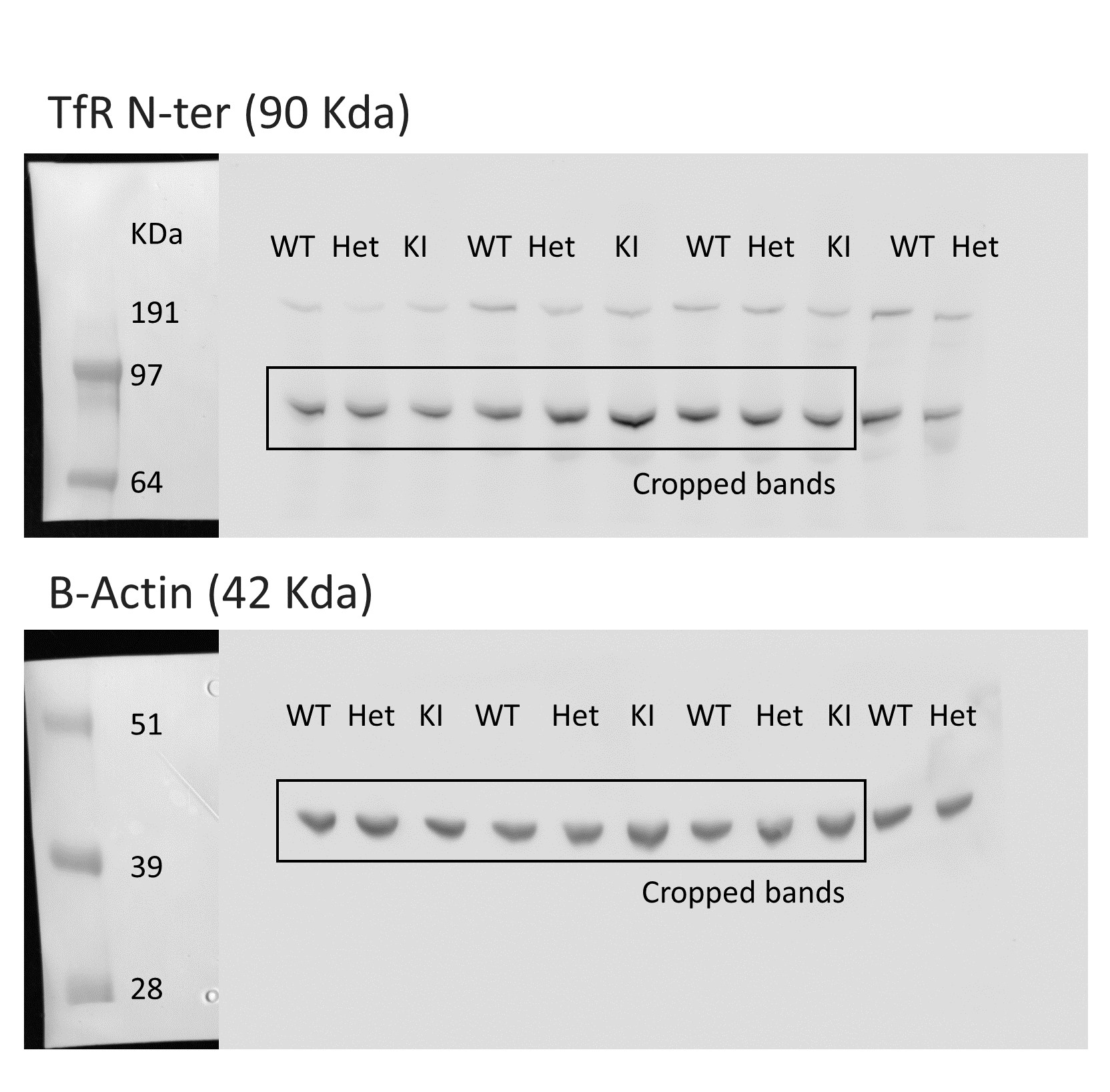

Supplement: Supplementary file 2 — Additional file 2: Figure S2. uncropped Western blot images from figure 3A. Blot was cut horizontally prior to staining to allow simultaneous staining for anti-TfR (upper panel) and B-Action (lower panel). [file 12987_2022_374_MOESM2_ESM.jpg]
